# Construction of ecological network in Qujing city based on MSPA and MCR models

**DOI:** 10.1038/s41598-024-60048-z

**Published:** 2024-04-29

**Authors:** Ji-zheng Qin, Ji-ping Dai, Song-hui Li, Jia-zhen Zhang, Jian-song Peng

**Affiliations:** 1 Southwest Forestry University, Kunming, 650224 Yunnan China; 2 Tengchong Forestry and Grassland Administration, Tengchong, 679100 Yunnan China; 3 Qujing Forestry and Grassland Administration, Qujing, 655000 Yunnan China

**Keywords:** MSPA-MCR model, Evaluation of ecological network connectivity, Geospatial data, Ecological network, Qilin district, Ecology, Forest ecology, Urban ecology

## Abstract

With the rapid advancement of urbanization and industrialization, ecological patches within cities and towns are fragmented and ecological corridors are cut off, regional ecological security is threatened and sustainable development is hindered. Building an ecological network that conforms to regional realities can connect fragmented patches, protect biodiversity and regional characteristics, and provide scientific reference for regional ecological protection and ecological network planning. By taking Qilin District, the main urban area of Qujing City as an example, and using geospatial data as the main data source, based on morphological spatial pattern analysis (MSPA) and minimum cumulative resistance (MCR), this study identified ecological source areas, extracted ecological corridors, and build & optimize ecological networks. (1) All landscape types are identified based on MSPA, the proportion of core area was the highest among all landscape types, which was 80.69%, combined with the connectivity evaluation, 14 important ecological source areas were selected. (2) 91 potential ecological corridors were extracted through MCR and gravity models, there were 16 important ones. (3) The network connectivity analysis method is used to calculate the α, β, and γ indexes of the ecological network before optimization, which were 2.36, 6.5, and 2.53, while after optimization, α, β and γ indices were 3.8, 9.5 and 3.5, respectively. The combined application of MSPA-MCR model and ecological network connectivity analysis evaluation is conducive to improving the structure and functionality of ecological network.

## Introduction

In recent years, with the rapid advancement of urbanization and industrialization, the expansion of construction land has been accelerating, resulting in the degradation of ecological environment, loss of species habitat, and serious landscape fragmentation in certain regions^1^. In this context, the rapidly expanding cities are confronted with various issues, such as the decline of habitat quality, the decrease of biodiversity and the threat of ecological security^2^. As the ecological governance technology advances, a large number of studies have proposed to relieve the contradiction between urban sprawl and habitat destruction by means of the construction of urban ecological network^3^. The construction of ecological network can not only promote the circulation of ecological materials and energy in the city, but also is of great significance for urban ecological spatial planning and the realization of regional sustainable development^4^.

Since the 1970s, the study of ecological network has attracted the attention of foreign scholars, who have made repeated attempts on the approaches and the model construction centering on ecological network from the macroscopic angle^5,6^. Currently, the structure of ecological network has become more mature, and the research on ecological network construction has begun to take shape: ecological source area identification, combined ecological resistance surface construction and potential ecological corridor extraction^7^. For the identification of ecological source areas, most existing studies take ecosystem services, ecological security, landscape connectivity, the importance of ecological function and other factors as the basis for evaluation, or directly select appropriate ecological patches based on patch area and attribute^8,9^. The combined resistance surface is mostly obtained based on the resistance assignment or the establishment of relevant evaluation systems. Some scholars have capitalized on nighttime light data, topographic potential index and geological hazard sensitivity to modify the combined resistance surface, in order to make the evaluation results more objective^10,11^. Minimum cumulative resistance (MCR) and circuit theory models are extensively utilized to extract potential ecological corridors, and important ecological corridors are identified based on gravity models^12–14^. For example: Du Xiaoyu et al.^13^ studied the ecological network construction in Yanqing District based on MSPA and MCR models; Huo Jingeng et al.^14^ used MSPA, MCR, gravity model and network structure evaluation to construct the ecological network of Zhengzhou metropolitan area. These research results not only provide certain theoretical support for local landscape planning and management, it also has certain practical significance in regulating ecological space and promoting landscape sustainability. However, there are few studies on plateau mountain forest cities in China, s ecological network.

As a national ecological demonstration zone, Qilin District in Qujing City is rich in forest resources. Nevertheless, with the focus on building a sub-central city in Yunnan Province and accelerating the pace of modern city construction, it is particularly important to create development opportunities and maintain ecological security in Qilin District. Taking Qilin District as an example, this study identified and extracted important ecological source areas through MSPA analysis, constructed combined resistance surfaces through the combination of landscape connectivity and landscape elements, extracted potential ecological corridors at all levels by MCR and gravity models, and added source areas, corridors and stepping stones through ecological network connectivity analysis, in order to optimize the ecological network structure. It provides scientific references for regional ecological protection and ecological network planning, and for ecological network construction of similar spatial scale cities.

### Overview of the study area

**Figure 1:**
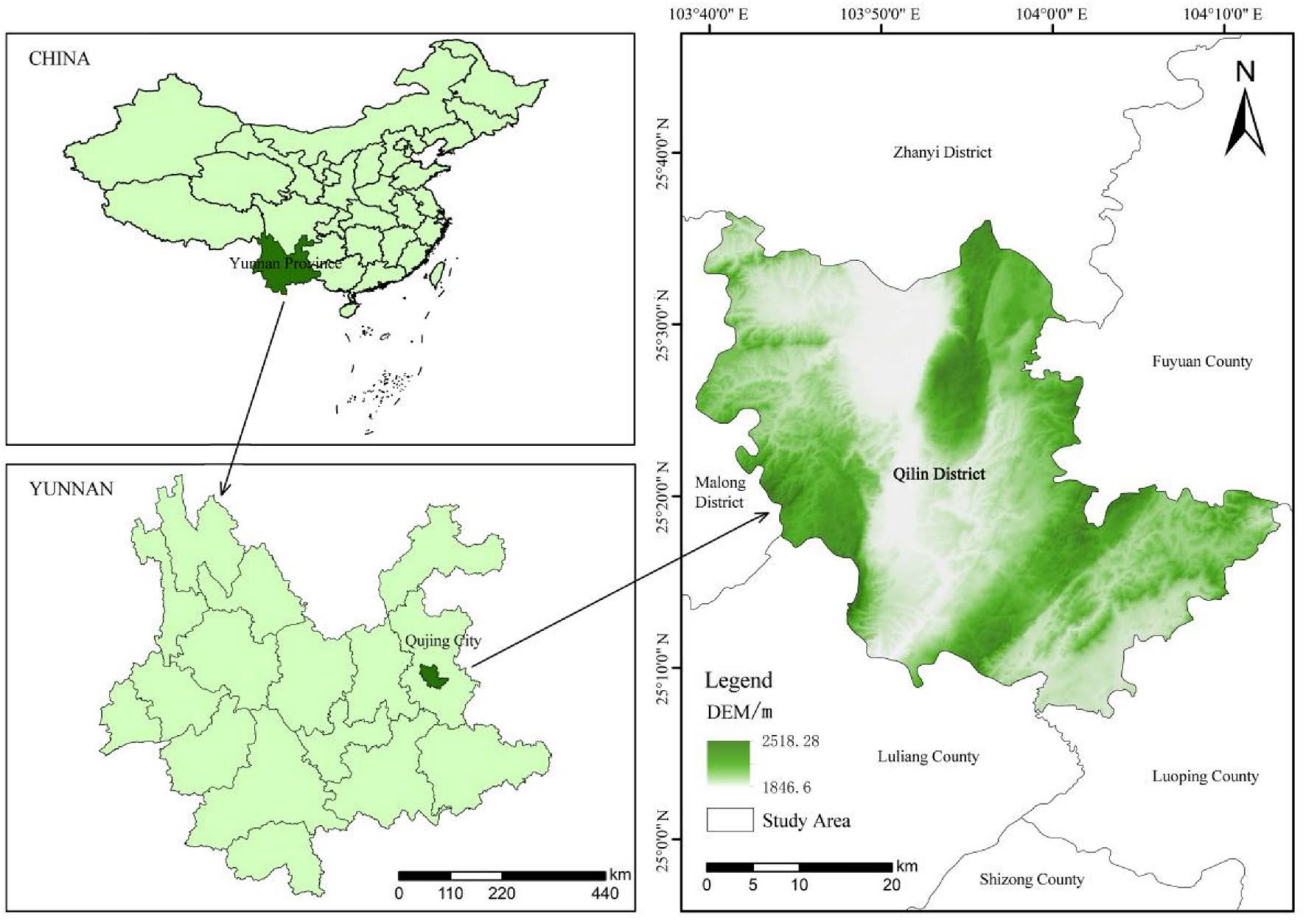
The study area.

Located at the source of the Pearl River, the third largest river in China, and the middle and upper reaches of the Jinsha River, Qujing City undertakes the construction of three major ecological security barriers in the Pearl River, Yangtze River basins and the western plateau. It is a typical plateau mountain forest city, the second largest economy and second largest city in Yunnan Province. Qilin District (25°08′ ~ 25°36′N, 103°10′ ~ 104°13′E), the headquarter of Qujing Municipal Committee and municipal government, is the main urban area of Qujing City. Situated in the east of Yunnan Province, the middle of the east Yunnan plateau and the upper reaches of Nanpan River, it is with an elevation of 1881 m and a total area of 1552.83km^2^ (Fig. 1). It has a north subtropical monsoon climate, with an average temperature of 16.4℃, annual rainfall of 802.3 mm, and 98% of the days with good air quality throughout the year. In 2023, Qilin District was named “Forest City of the Province” (https://lcj.yn.gov.cn/html/2023/zuixindongtai_0721/69389.html) by the Forestry and Grassland Bureau of Yunnan Province. There are 4 county-level nature reserves in the district, with a forest coverage rate of 48.8%.

### Data sources and research methods

#### Data sources and processing

The data used in this study mainly included administrative division, DEM, slope, land use, NDVI etc. The administrative vector data was derived from BIGEMAP map downloader (http://www.bigemap.com/), and DEM digital elevation data from geospatial data cloud (https://www.gscloud.cn, resolution 30 m × 30 m). Based on DEM and ArcGIS10.7, the slope data was processed through inlaying, mask extraction and projection, and finally obtained by means of 3D Analyst tool, for instance, raster surface. On the basis of the Landsat 8OLI_TIRS remote sensing image (https://www.usgs.gov, resolution 30 m × 30 m, cloudcover 1.13%) in March 2021, the land use data was extracted by the interpretation of ENVI5.3 supervised classification, in combination with visual observation. Combined with the field investigation, the data interpreted was revised and proofread repeatedly. Finally, land use types were generated based on the ArcGIS10.7, including 6 types of woodland, water body, grassland, cultivated land, construction land and other land according to the current national classification standard^15^, and the confusion matrix was used to verify the accuracy. The overall accuracy reached 86.30% and the Kappa coefficient was 0.8, which met the needs of this research. The NDVI data was based on Landsat8OLI_TIRS remote sensing image (https://www.gscloud.cn, resolution 30 m × 30 m, cloudcover 1.13) in March 2021 and calculated by ENVI5.3.

### Research methods

#### Analysis of landscape patterns

MSPA, proposed by Vogt et al.^16^, is an image processing method that relies on the principle of mathematical morphology to measure, identify and segment the spatial pattern of raster images. Referring to relevant studies^17^ and based on land use data, woodland was extracted as foreground data (assigned value 2), and the rest was background data (assigned value 1). Based on the Guidos Toolbox software, eight-neighborhood image thinning analysis was applied to analyze the binary raster 8-bit Tiff data, and 7 non-overlapping landscape elements, including core area, island, pore, and edge area, were obtained, so that structural elements such as core area and corridor number were directly displayed in the results. Among them, the core area was the best choice for species habitat due to its large area, small degree of fragmentation, and complete shape. Consequently, the core area was selected as the alternative source area. According to the Gudios Toolbox (GuidosToolbox_Manual (europa.eu)), the threshold value of the core area was 17/117.

#### Identification of ecological source areas

Important ecological source areas are areas where material exchange and energy flow are sufficient, and their accurate identification is the key to the subsequent construction of ecological network. This study took patch area size and landscape connectivity index into account in a comprehensive manner to select important source areas^18^. Two landscape connectivity indices, integral index of connectivity (IIC) and probability index of connectivity (PC), can be used to reflect the connectivity of landscape patches and calculate the importance value of patches (dPC) to landscape connectivity^19,20^. The calculation formula is as follows:

1 $$IIC = \frac{{\sum\nolimits_{i = 1}^{n} {\sum\nolimits_{j = 1}^{n} {\left( {\frac{{a_{i} \cdot a_{j} }}{{1 + nl_{ij} }}} \right)} } }}{{A_{{}}^{2} }}$$

2 $$PC = \frac{{\sum\nolimits_{i = 1}^{n} {\sum\nolimits_{j = 1}^{n} {a_{i} \cdot a_{j} \cdot p_{ij}^{ * } } } }}{{A_{{}}^{2} }}\left( {0 < PC \leqq 1} \right)$$

3 $$dPC = \frac{{PC - PC_{remove} }}{PC} \times 100\%$$

where: n is the total number of patches, a is the patch area, nl_ij_ is the number of connections between patches i and j, and P*_ij_ is the maximum probability of species migration path between patches i and j. A is the total landscape area, and PC_remove_ is the landscape connectivity of remaining patches after randomly removing patch i.

#### Construction of resistance surface

A resistance surface consists of one or more resistance layers. Resistance factors such as land use type, DEM, slope, distance from water body, distance from road and distance from residential site are often used in relevant researches, and different combinations are available depending on research needs^21,22^. Combined with previous studies and the actual situation of the study area, the resistance factors of DEM, slope, NDVI and land use type were selected, and the assigned value of resistance was between 1 and 5. The smaller the value, the smaller the landscape resistance and the higher the suitability of biological activities. And use the Analytic Hierarchy Process (AHP) to assign weights of resistance factors at all levels, the weights of resistance factors at all levels were set as 0.18, 0.15, 0.31 and 0.36 respectively^23,24^ (Table 1). Finally, the combined resistance surface was weighted by the grid calculator and used as the cost data of the MCR model.

**Table 1:** Resistance factor assignment and weight.

Resistance factor	Grading standard	Resistance value	Weight
DEM(m)	≤ 1927	1	0.18
	1927–2019	2	
	2019–2116	3	
	2116–2224	4	
	> 2224	5	
Slope(°)	≤ 4	1	0.15
	4–8	2	
	8–12	3	
	12–19	4	
	> 19	5	
NDVI	> 0.39	1	0.31
	0.22–0.39	2	
	0.05–0.22	3	
	≤ 0.05	4	
Land use type	forest land	1	0.36
	grassland、cultivated land	2	
	other land	3	
	water body	4	
	construction land	5	

#### Extraction of ecological corridors

As the framework of ecological network, corridor reflects the possibility and tendency of species movement between source areas, and plays a role in promoting species diffusion and strengthening ecological functions^25,26^. Guided by species preference, the MCR model is used to obtain the lowest-cost path by calculating the cost distance between the source area and the target source area, so as to determine the best path of biological migration and diffusion as an ecological corridor^27^. The calculation formula is as follows:

4 $$MCR = f_{{{\text{min}}}} \sum\limits_{j = n}^{i = m} {(D_{ij} \times R_{i} )}$$

where: f is an unknown increasing function, m is the number of landscape unit i, and n is the number of ecological source j. D_ij_ is the spatial distance from j to i, while R_i_ is the cmbined resistance coefficient of i to the movement of a certain species.

The gravity model can quantitatively evaluate the intensity of the interaction between patches, and the greater the value of the interaction force, the more important the potential corridor between the two in the regional ecosystem^28^. The calculation formula is as below:

5 $$G_{ij} = \frac{{L_{\max }^{2} \ln (S_{i} )\ln (S_{j} )}}{{L_{{{\text{ij}}}}^{2} P_{i} P_{j} }}$$

where: G_ij_ is the interaction force between patch i and j, L_max_ is the maximum resistance value of all corridors, and S_i_ is the area of patch i. L_ij_ is the cumulative resistance value of the corridor between i and j, and P_i_ is the resistance value of patch i.

#### Analysis of Network Connectivity

Connectivity can reflect the connectivity degree of ecological network in a quantitative fashion^29^. In this study, ecological network connectivity analysis approach was utilized to quantitatively describe the closure, complexity and connectivity of the potential ecological network structure constructed, by calculating three indexes of network closure (α), line point rate (β) and network connectivity (γ)^30,31^. The calculation formula is as follows:

6 $$\alpha = \frac{L - V + 1}{{2V + 5}}$$

7 $$\beta = \frac{L}{V}$$

8 $$\gamma = \frac{L}{{3\left( {V - 2} \right)}}$$

where: L is the number of corridors and V is the number of nodes.

## Results and analysis

### Analysis of MSPA landscape patterns

**Figure 2:**
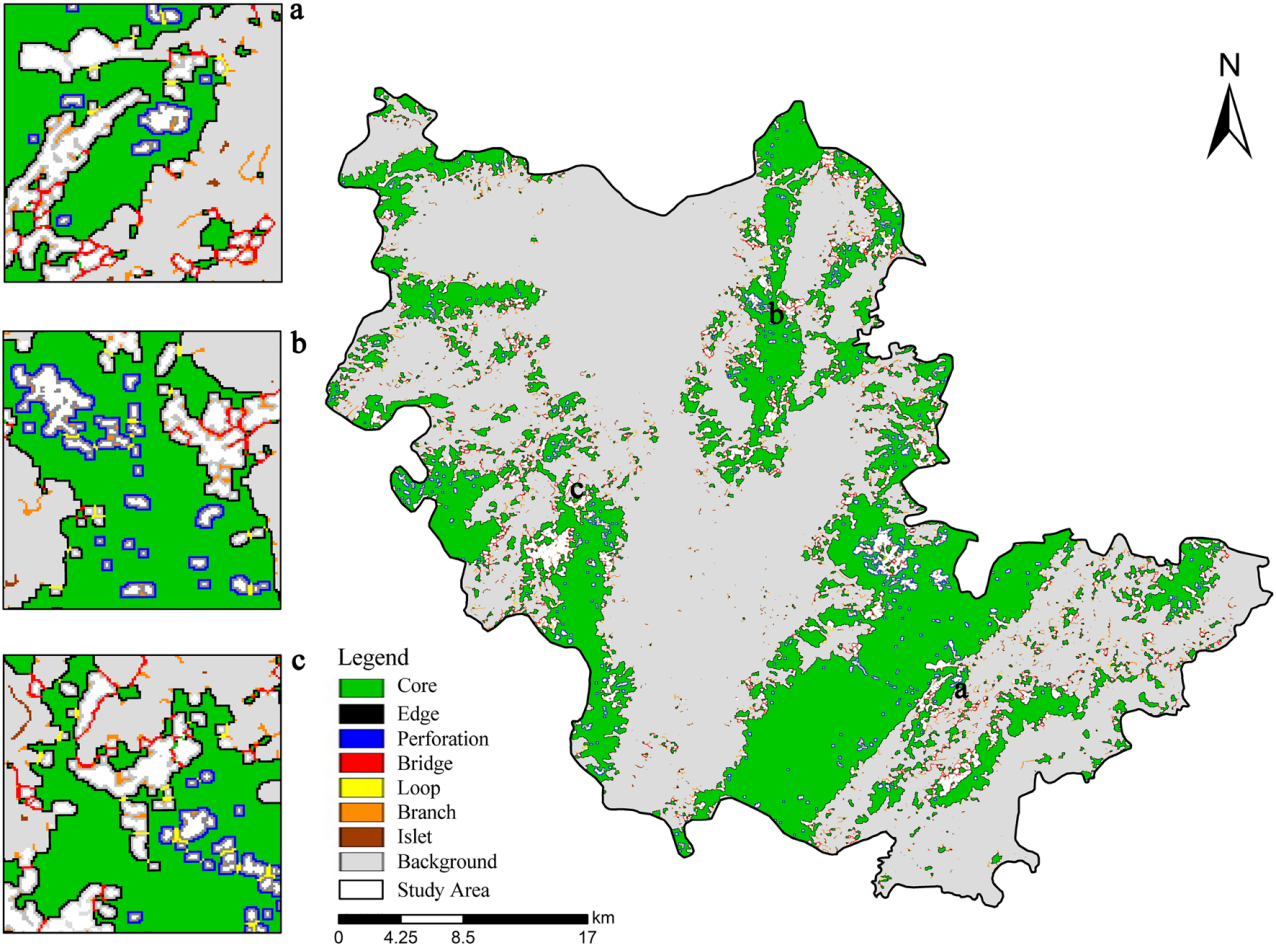
MSPA landscape analysis.

As shown in Fig. 2 and Table 2, there were7 landscape types analyzed based on MSPA, with a total area of 496.46km^2^, accounting for 31.97% of the whole study area. The core area was 400.58km^2^, occupying 80.69% of the ecological space area, with the largest proportion, mainly distributed in the west, southeast and northeast of the study area, with large patch area and strong stability. However, the lack of connectivity among the three core patches was not conducive to the material exchange and energy flow among the patches. The proportion of marginal areas and pores was 12.31% and 2.10%, respectively, indicating that there was a serious issue of patch fragmentation in the study area, and it was necessary to strengthen the landscape connectivity between patches in the core area. As the corridor connecting patches in the ecological network, the bridge area was distributed near the core patch, accounting for 1.52%, displaying that there were few structural corridors in the landscape pattern of the study area, not beneficial to species migration, and corridor optimization was called for. As a temporary habitat for the flow of ecological factors, isolated islands scattered in the study area took up only 0.53%. The stability of the ecological network can be enhanced by adding stepping stones in the future. The remaining landscape types were loops (0.43%) and branches (2.42%).

**Table 2:** MSPA classified statistics.

Landscape type	Area (km^2^)	Proportion of ecological land area (%)	Proportion of total area (%)
Core	400.58	80.69	25.80
Islet	2.64	0.53	0.17
Perforation	10.43	2.10	0.67
Edge	61.09	12.31	3.93
Loop	2.15	0.43	0.14
Bridge	7.53	1.52	0.48
Branch	12.04	2.42	0.78
Total	496.46	100.00	31.97

### Evaluation of the connectivity of important ecological source areas

Referring to relevant studies^32,33^, 26 source areas with the largest patch area in the core area were chosen for connectivity analysis. Based on Conefor 2.6 software, the patch connectivity distance threshold was set as 500 m, 1000 m, 1500 m, 2000 m and 2500 m, respectively. In order to be comparable with the IIC results, the probability of patch connectivity was set to 0.5. It can be concluded from the comparison that if the distance threshold is too large, some large patches will be segmented, while some small ones will disappear. As the threshold changes, the dIIC and dPC values also change. Within a certain range, the ranking of the importance of patches reflected by dIIC and dPC is relatively consistent. In the end, the distance threshold was set to 500 m, and 14 patches with dPC > 0.5 were defined as important ecological source areas (Table 3).

**Table 3:** Importance value index of important ecological source areas.

Ranking	Source area no.	Area/km^2^	dIIC	dPC
1	14	165.90	86.29	85.52
2	8	29.04	4.88	5.71
3	13	39.74	4.72	4.57
4	7	5.22	2.70	3.87
5	2	12.21	2.39	3.17
6	12	1.77	0.91	1.31
7	10	20.87	1.30	1.26
8	1	2.06	0.77	0.95
9	11	1.17	0.60	0.85
10	4	4.34	0.63	0.75
11	9	2.95	0.41	0.65
12	6	4.17	0.59	0.61
13	3	13.34	0.53	0.52
14	5	12.05	0.50	0.50

**Figure 3:**
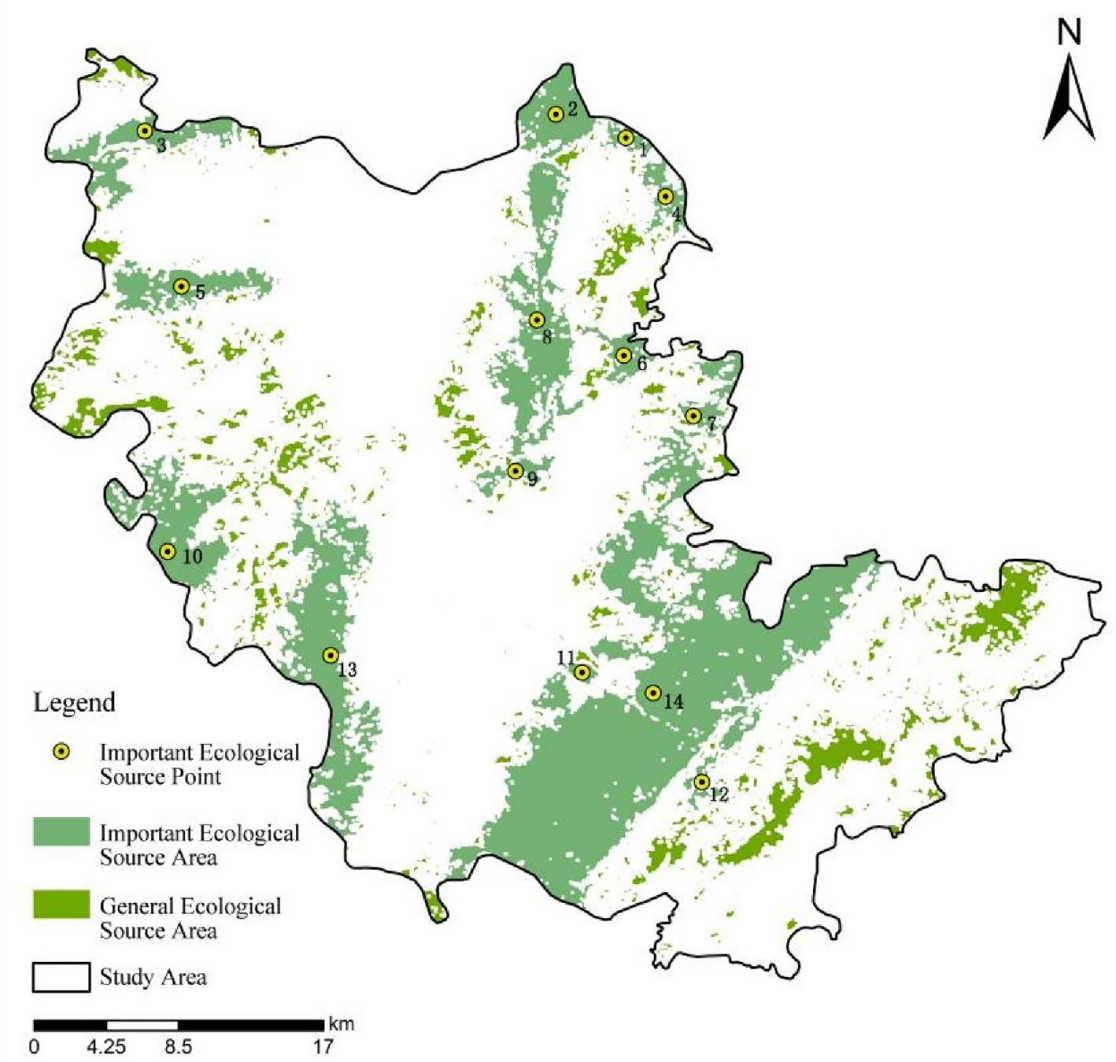
Distribution of important ecological source areas.

As could be seen from Fig. 3, important ecological source areas were mainly distributed in the western, northwestern, northeastern and southeastern parts of the study area, with the northwestern part sparsely distributed. Important ecological areas, such as nature reserves, forest parks and natural forests, were identified, which had high vegetation coverage, rich biodiversity, high ecological value and could sufficiently represent the ecological level of the study area. While the connectivity between the source areas was poor. Table 3 showed that ecological source area No. 14, the largest one with the best landscape connectivity, was the core of ecological circulation in the whole study area. The connectivity of ecological source areas in northeast China was strong, beneficial to species migration and flow. In other regions, the ecological source area was small, the space more dispersed and the landscape connectivity poorer. In order to promote the overall ecological coordination in the study area, small and medium-sized ecological nodes could be appropriately established in the central and southern regions to optimize the balance of regional ecosystems.

### Construction of combined resistance surface

**Figure 4:**
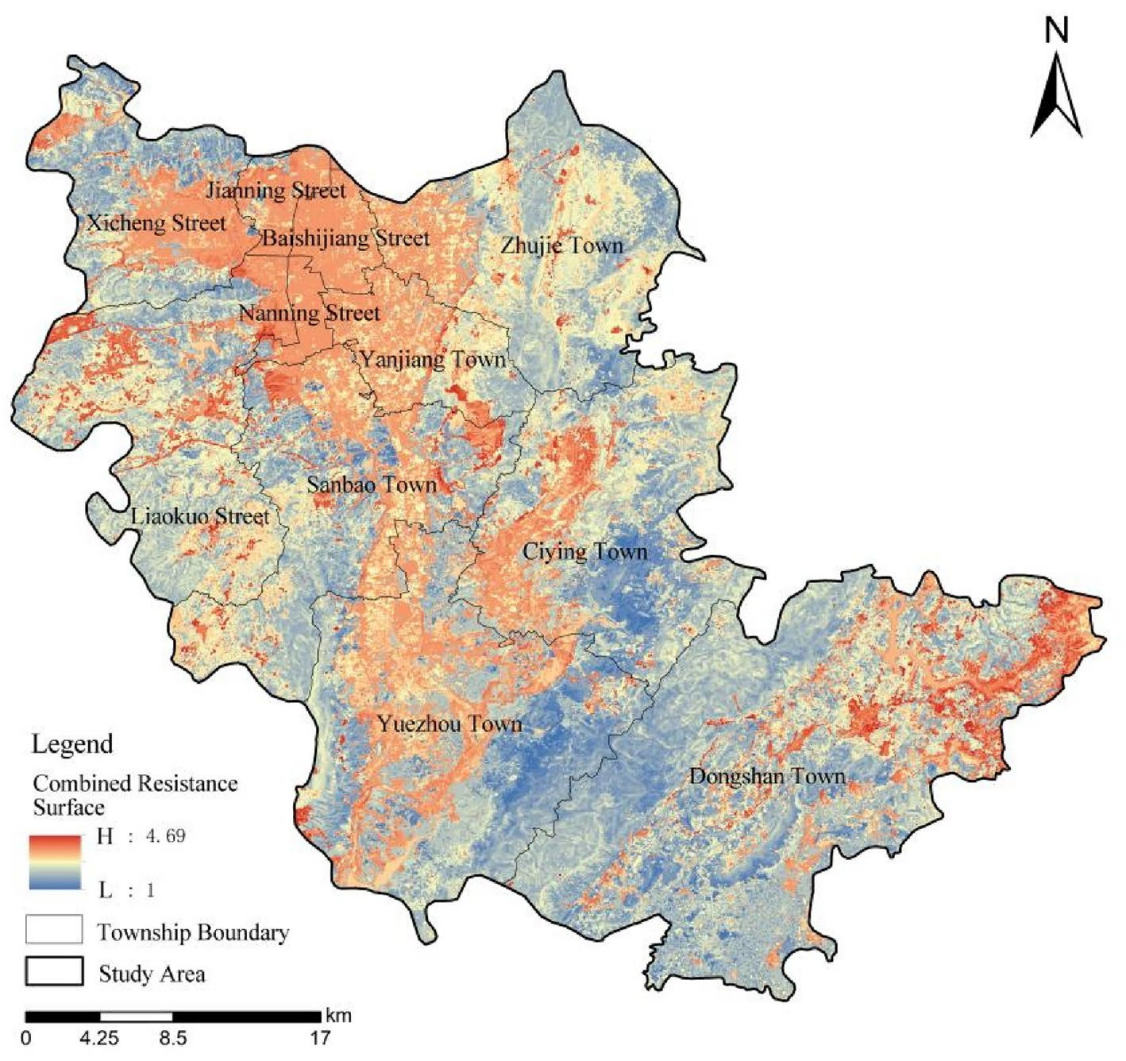
Combined resistance surface.

As shown in Fig. 4, the resistance values in the west, northwest, northeast and southeast of the study area were small, while there were also some areas with high resistance values, such as Xicheng Street, Liaokuo Street, Sanbao Town and Dongshan Town. The areas with high resistance were mainly located in the central and northern part of the study area, including Jianning Street, Baishijiang Street, Nanning Street, Yanjiang Town, etc. This was because these areas were under the influence of human activities, such as construction land and cultivated land, and almost all of them were in urban and township built-up areas, which to a large extent hindered the flow of ecosystem and ecological information. Some natural forestlands with high ecological value could be protected by setting up shelterbelts, and other measures, for example, adding stepping stones in other areas, could be taken to alleviate ecological disconnection.

### Extraction and analysis of important ecological corridors

**Figure 5:**
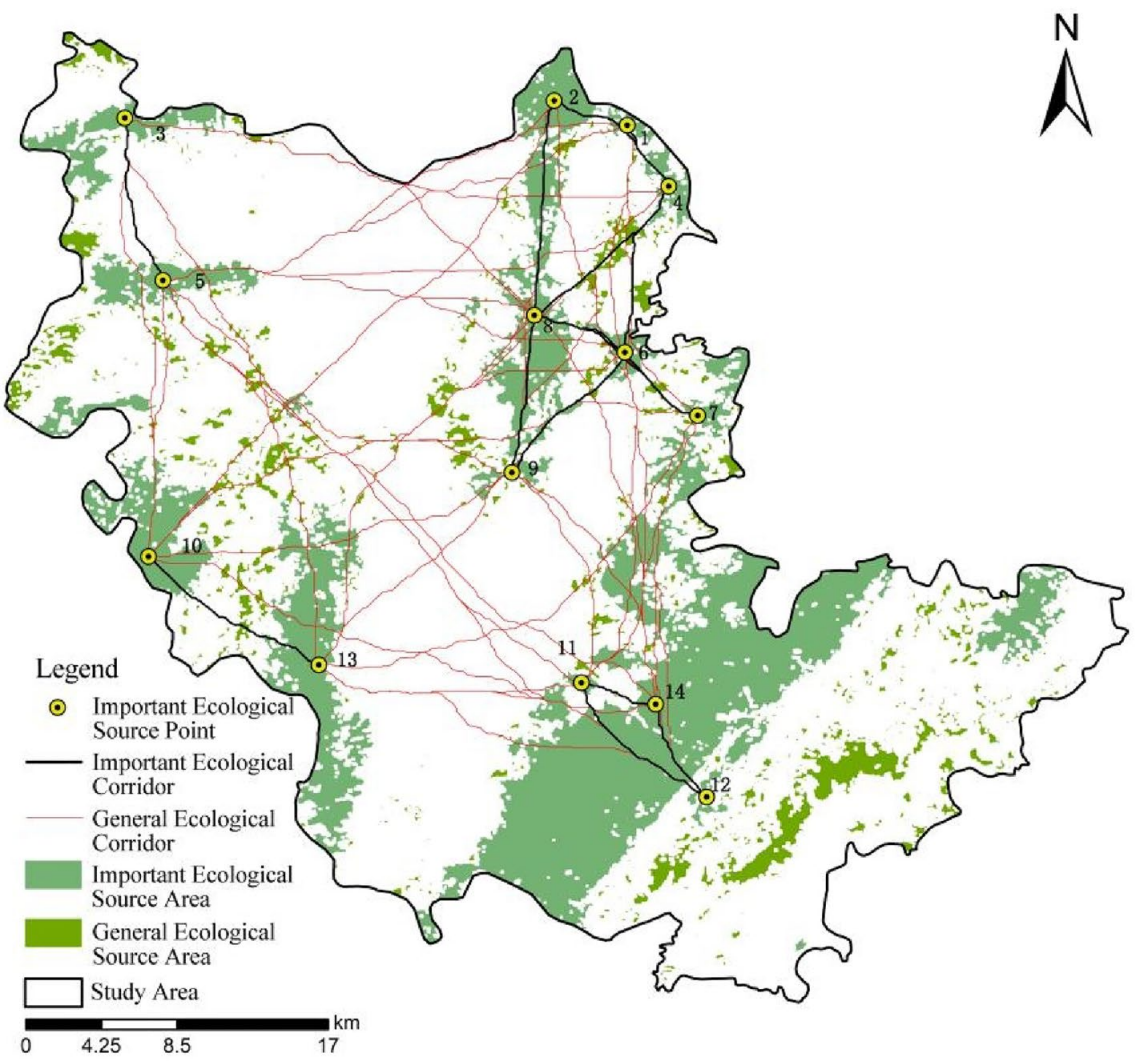
Distribution of important ecological corridors.

According to the above analysis, 91 potential ecological corridors were acquired by calculating the minimum path from source patch to target patch based on the cost distance and cost path through Spatial Analysis tool. On the basis of the gravity model, the interaction matrix among 14 important ecological source areas was constructed to quantitatively analyze and evaluate the relative importance of potential corridors (Table 4). The 16 corridors with the greatest interaction force were extracted as important corridors, with the redundant corridors deleted, so as to obtain the important ecological corridors in the study area (Fig. 5).

**Table 4:** Interaction matrix among important ecological source areas.

No. of source area	1	2	3	4	5	6	7	8	9	10	11	12	13	14
1	0	8489.33	138.48	9745.10	125.26	818.08	369.79	880.66	294.24	87.05	141.86	102.30	109.85	178.78
2		0	188.39	2573.75	166.94	653.54	318.17	1090.57	333.71	100.11	134.95	99.46	126.19	170.28
3			0	129.45	1307.14	124.01	82.40	172.12	127.33	210.40	74.61	54.89	136.19	80.36
4				0	119.92	1453.01	563.47	1517.94	411.68	106.48	182.85	127.61	135.80	230.03
5					0	151.93	103.48	222.86	231.83	500.47	112.72	76.05	247.56	115.59
6						0	4587.85	5622.96	1732.10	160.34	459.74	265.66	246.14	572.02
7							0	1219.84	809.38	111.89	595.34	313.10	175.35	740.85
8								0	1825.32	205.68	302.63	191.44	277.78	375.69
9									0	267.09	678.82	312.37	543.44	728.22
10										0	176.53	113.48	1000.03	175.83
11											0	1983.16	571.12	9420.44
12												0	293.36	5737.95
13													0	487.20
14														0

As shown in Table 4, the interaction between source area 1 and 4 was the strongest, indicating that the resistance of biological migration between source areas was the least and the degree of connectivity was the highest. Taking a prominent position in the ecological network, corridors should be strengthened in terms of control and protection. The distance between source area 3 and 12 was the furthest, and the interaction between the two was the weakest, demonstrating that the landscape resistance of the ecological corridor between the source areas was relatively large and the connectivity was weak, which could not fulfill the requirements for species migration and energy diffusion, undermining the species richness and biodiversity of the study area. Optimization was needed in this regard.

From Fig. 5, it could be seen that important ecological corridors were mainly distributed in parts of the west, northwest, northeast and southeast. The connectivity was strongest in the northeast, majorly located in Wutai Mountain and Langmushan Mountain county-level nature reserves, but the four parts were relatively independent and had poor connectivity with each other. It followed then that the ecological network in the study area was not sound enough to form complete network connection. Ecological nodes could be supplemented on the basis of the extracted corridors to promote the material exchange and biological exchange in the study area.

### Analysis of ecological corridor network connectivity

**Figure 6:**
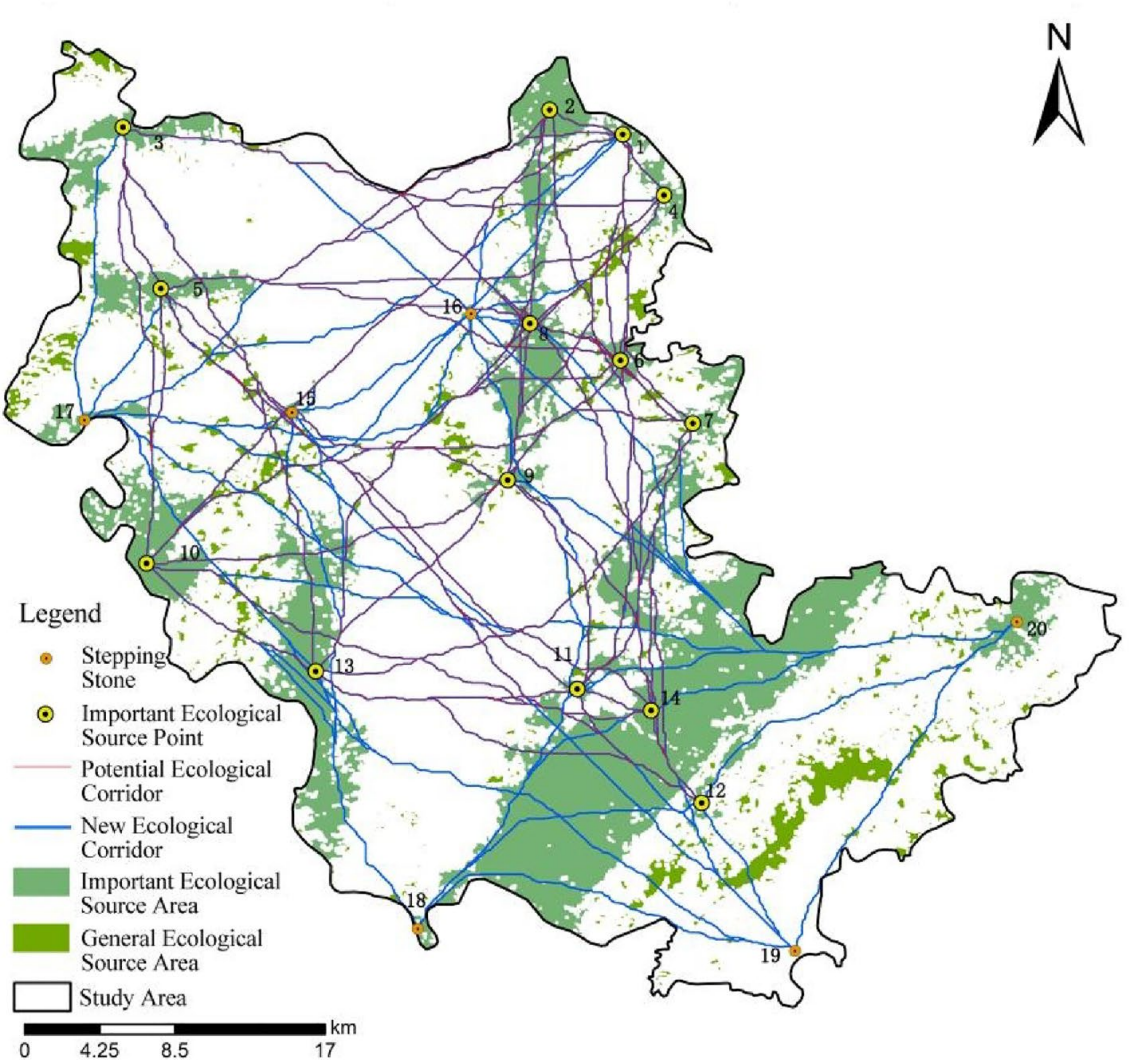
Optimized ecological network system of the study area.

By calculating the ecological network connectivity, it was observed that the α, β and γ indices before the optimization of ecological network in the study area were 2.36, 6.5 and 2.53, respectively. As shown in Fig. 6, according to the importance degree and location distribution of general ecological source areas, this study selected 6 complementary important ecological source areas as stepping stone patches, including 1 in the south, 1 in the northwest, 2 in the southeast, and 2 in the middle. Based on the supplementary calculation of the original important ecological source areas, 99 new planned ecological corridors were obtained. After optimization, there were 20 important ecological source areas and 190 ecological corridors in the study area. The α, β and γ indices of the ecological network after optimization were 3.8, 9.5 and 3.5, respectively, which greatly improved the connectivity of the ecological network and optimized the ecological network. In the future planning and construction, more attention could be attached to stepping stone patches, and the ecological network system of the study area could be improved by constructing new ecological corridors.

## Discussions and conclusion

### Discussions

Based on MSPA-MCR model analysis and ecological network connectivity analysis evaluation, this study optimized the ecological network of the study area, which has certain guiding significance for the future planning and construction of the study area. Whereas, the method of constructing and optimizing ecological network is still under exploration, and following shortcomings remain: In the identification of ecological source area, MSPA is very sensitive to the pixel size of the study area, and the influence of different particle sizes and edge widths on the ecological network pattern has not been taken into account. In the analysis of landscape connectivity index, the setting of patch connectivity distance threshold and patch connectivity probability have been verified to some extent, but still need to be further analyzed. Regarding the selection of ecological source area and ecological corridor, the influence of quantity on the results needs more attention. The construction of resistance surface is a key step of ecological network. The selection and assignment of resistance factors exert an important influence on the formation of corridors and the results of ecological network, and more attention in this regard is called for in future research.

The construction of stepping stone patches can serve as a resting place for organisms, improve the connectivity of ecological corridors and the integrity and stability of ecological networks. However, in future development, it is necessary to strengthen ecological restoration, improve the vegetation coverage of the source area itself, and at the same time strengthen the resource integration between the source area and surrounding source areas, edges and isolated islands, advocate afforestation and other methods to expand the area of the source area and create connectivity the entire ecological core reduces landscape fragmentation and islanding, and further optimizes the regional ecological network.

## Conclusion

Taking Qilin District as the study area, this study constructed the potential ecological network by MSPA-MCR and gravity model and further optimized the ecological network through ecological network connectivity analysis evaluation. The main conclusions are as follows:

1. Seven landscape types are identified, of which the core area is the largest, accounting for 80.69% of the ecological space area. Based on the selection of dPC connectivity index and patch area, 14 important ecological sources are screened. The main distribution area contains important ecological areas such as nature reserves, forest parks and natural forests, which possess high ecological value, but the connectivity of ecological source areas is poor, proper distribution of important source areas not formed.
2. The resistance values of urban built-up area and township built-up area are higher, while the resistance values of other areas are lower. A total of 91 potential ecological corridors and 16 important ecological corridors are extracted. Important corridors are mainly distributed in the west, northwest, northeast and southeast regions, and some regional corridors are missing in the study area. Due to the influence of human activities including construction land and cultivated land, the spatial distribution of ecological network is unbalanced, which obstructs the flow of ecosystem and ecological information to a large extent.
3. By optimizing the spatial structure of the ecological network, a total of 6 stepping stone patches are added due to the loss of the ecological corridors in the southern, northwestern, southeastern and central regions of the region. Comparing the network structure before and after optimization, it can be observed that α, β and γ indices increase by 1.44, 3 and 0.97, respectively. This displays that the overall distribution of the optimized ecological network is more balanced, the complexity of ecological network connections is immensely improved, and a more stable and rich ecological network pattern is formed.

## Data Availability

The data that support the findings of this study are available from the corresponding author, upon reasonable request.
